# Functional outcomes and complications of open elbow dislocations

**DOI:** 10.1007/s11678-018-0466-0

**Published:** 2018-06-11

**Authors:** Marc Schnetzke, Felix Porschke, Ulrich Kneser, Stefan Studier-Fischer, Paul-Alfred Grützner, Thorsten Guehring

**Affiliations:** 1 BG Trauma Center Ludwigshafen, Clinic for Trauma and Orthopaedic Surgery, University of Heidelberg, Ludwig-Guttmann-Str. 13, 67071 Ludwigshafen on the Rhine, Germany; 2 BG Trauma Center Ludwigshafen, Department of Plastic, Reconstructive and Hand Surgery, Burn Care Centre, Department of Plastic Surgery, University of Heidelberg, Ludwigshafen on the Rhine, Germany

**Keywords:** Elbow joint, Joint instability, Soft tissue injuries, Revision surgery, Treatment outcome, Ellenbogengelenk, Gelenkinstabilität, Weichteilschaden, Revisionsoperation, Therapieergebnis

## Abstract

**Background:**

The current study investigated the clinical outcome of open elbow dislocations, focusing on the influence of associated soft tissue and bone injury.

**Patients and methods:**

From October 2008 to August 2015, 230 patients with elbow dislocations were treated at the study center. Our retrospective study comprised 21 cases of open elbow dislocations. The mean age of patients was 49 years (20–83 years); there were six (29%) female and 15 (71%) male patients. The range of motion (ROM) of the injured and uninjured elbow was measured, and the Mayo Elbow Performance Score (MEPS), Mayo Wrist Score (MWS), and Disability of Arm, Shoulder and Hand (DASH) score were assessed. Complications and revision surgeries were recorded. The influence of the severity of soft tissue injury (I°/II° open vs. III° open) and type of dislocation (simple vs. complex) was evaluated.

**Results:**

After a 57-month follow-up (range, 24–98 months), the mean DASH score was 20 ± 15, the MEPS was 82 ± 11, and the MWS was 74 ± 22. The ROM of the injured elbow was significantly decreased compared with the uninjured one (arc of ulnohumeral motion: 104° vs. 137°; *p* = 0.001). Patients with I°/II° open elbow dislocations had a better clinical outcome according to the MEPS (86 ± 11 vs. 76 ± 9; *p* = 0.045) and a comparable outcome according to the DASH score (19 ± 18 vs. 21 ± 9; *p* = 0.238). In all, 11 patients (52%) had postoperative complications and 11 patients underwent at least one revision surgery. Complex elbow dislocations had significantly more complications and revision surgeries than simple dislocations (77% vs. 13%; *p* = 0.008).

**Conclusion:**

Favorable clinical outcomes can be achieved after treatment of open elbow dislocations. These injuries are prone to neurovascular damage and complex dislocations are linked to high rates of complications and revision surgeries.

## Zusammenfassung

**Hintergrund:**

Im Rahmen der vorliegenden Studie sollte der Einfluss des Weichteilschadens auf das klinische Ergebnis nach offener Ellenbogenluxation untersucht werden.

**Material und Methoden:**

Von Oktober 2008 bis August 2015 wurden insgesamt 230 Patienten mit Ellenbogenluxation behandelt. Diese retrospektive Studie umfasst 21 Fälle von offenen Ellenbogenluxationen. Das Durchschnittsalter der Patienten betrug 49 Jahre alt (20–83 Jahre), 6 Patienten waren weiblich (29%), 15 männlich (71%). Das Bewegungsausmaß des verletzten und unverletzten Ellenbogens wurde erhoben und das funktionelle Ergebnis u. a. mittels Mayo Elbow Performance Score (MEPS), Mayo Wrist Score (MWS) und dem Disability of Arm, Shoulder and Hand (DASH) Score erfasst. Zusätzlich wurden Komplikationen und Revisionsoperationen aufgezeichnet. Der Einfluss des Weichteilschadens (I°/II° offen vs. III° offen) und des Luxationstyps (einfach vs. komplex) auf das klinische Ergebnis wurde analysiert.

**Ergebnisse:**

Nach einem durchschnittlichen Nachuntersuchungszeitraum von 57 Monaten (24–98 Monate) betrug der DASH-Score 20 ± 15 Punkte, der MEPS 82 ± 11 Punkte und der MWS 75 ± 23 Punkte. Die Beweglichkeit am betroffenen Ellenbogengelenk war im Vergleich zur gesunden Gegenseite signifikant schlechter (Extension/Flexion: 104° vs. 137°; *p* = 0,001). Das klinische Ergebnis von Patienten mit offener Luxation vom Grad 1 oder 2 war signifikant besser, gemessen am MEPS (86 ± 11 vs. 76 ± 9; *p* = 0,045), jedoch ohne Unterschied bezüglich des DASH-Scores (19 ± 18 vs. 21 ± 9; *p* = 0,238). Bei 11 Patienten (52 %) trat eine postoperative Komplikation auf, und 11 Patienten wurden mindestens einmal operativ revidiert. Komplexe Luxationen wiesen eine signifikant höhere Rate an Komplikationen und Revisionsoperationen auf (77 % vs. 13 %; *p* = 0,008).

**Schlussfolgerung:**

Offene Ellenbogenluxationen können mit einem zufriedenstellenden klinischen Ergebnis einhergehen. Insbesondere komplexe offene Ellenbogenluxationen sind jedoch sehr komplikationsbehaftet, wobei neurovaskuläre Komplikationen am häufigsten auftreten.

Open elbow dislocations are a rare variant of elbow dislocations and the treatment is challenging. In the current study, we analyze the clinical outcome of open elbow dislocations and determine the influence of associated soft tissue and bone injury.

## Introduction

The overall incidence of traumatic elbow dislocations is 6–13/100,000 cases per year [14]. Elbow dislocations can be divided into simple dislocations with pure ligamentous injuries and complex dislocations with concomitant fractures of the radial head, olecranon, coronoid, or the distal humerus [9]. The majority of simple elbow dislocations are stable after reduction and can be treated by functional rehabilitation of the elbow [3, 6, 12]. Complex elbow instabilities are characterized by periarticular fractures with damage to the articular surface and require surgical repair [8].

Open elbow dislocations represent a rare variant of this injury and result from a high-energy trauma mechanism [2]. The primary goal of treatment in these cases is reconstruction of a congruent and stable joint [13]. Current knowledge on the management and expected functional outcome in open elbow dislocations is based on a limited number of reports that included a small number of patients [1, 2, 7]. The aim of the current study was to investigate the clinical outcome of open elbow dislocations in a much larger number of patients with particular focus on the influence of the associated soft tissue and bone injuries on clinical outcome.

## Methods

### Patients

**Fig. 1:**
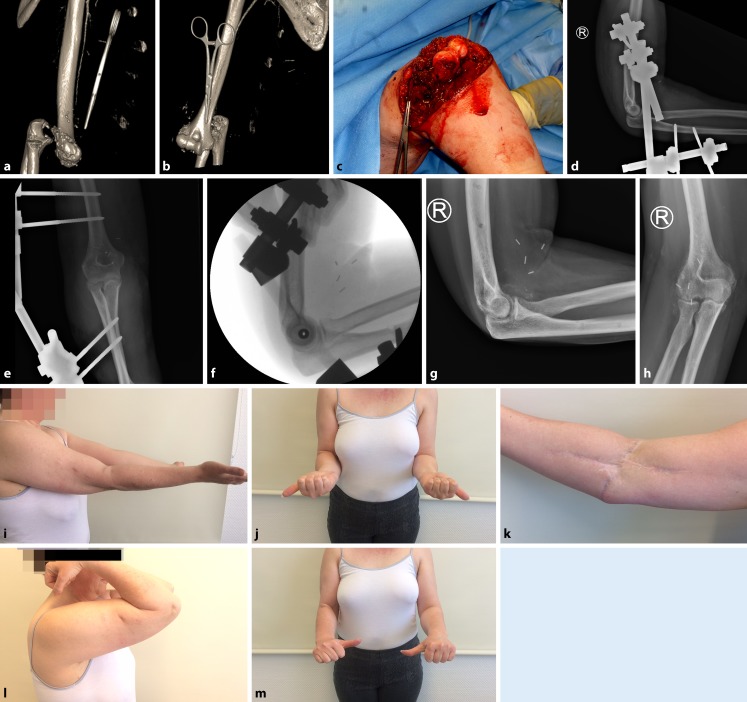
Case 1 (patient no. 6). **a**–**c **A 50-year-old woman fell from a horse on her right arm and sustained an open simple elbow dislocation with primary disruption of the brachial artery (vessel clamp attached to stop the bleeding, artifacts around the clamp). **d**, **e **During primary surgical treatment, the brachial artery was reconstructed using an autologous vein graft and the elbow was stabilized with an external fixator. **f **After 17 days, the fixator was mounted to a hinged elbow external fixator for another 4 weeks. **g**–**m **At the 24-month follow-up, there was a good clinical outcome (MEPS 85, VAS_r_ 0) with an ulnohumeral arc of motion of 120° and arc of pronation/supination of 160°. **g **Periarticular ossifications were noted on the lateral radiograph that did not affect the clinical outcome

This retrospective study was enrolled at a level I trauma center after approval by the local ethics committee (837.084.14[9323-F]). The study inclusion criteria were age ≥18 years, written informed consent, simple or complex open elbow dislocation, and minimum follow-up of 24 months. Between January 2008 and August 2015, a total of 230 elbow dislocations were treated at the study institution, of which 24 (10%) were classified as open dislocation. Two patients could not be reached for final examination and one patient died for unrelated reasons. Finally, 21 patients (88%) with open elbow dislocations were included in the study (Table 1). The mean age of the study population was 49 years (range, 20–83 years). Six patients (29%) were female and 15 (71%) were male. The right side was injured in seven patients (33%) and the left side in 14 patients (67%); the injury affected the dominant side in ten patients (48%). In 13 patients (62%) the injury was classified as complex elbow dislocation and in eight patients (38%) as simple elbow dislocation with pure ligamentous injury. The soft tissue damage in both open fracture dislocations and in dislocations with pure ligamentous injury was graded according to the classification system of Gustilo and Anderson [5]. The elbow dislocation was graded as I° open in four patients (19%), II° open in nine patients (43%), and III° open in eight patients (38%). Three patients sustained neurovascular injury, two patients had a primary injury of the radial nerve (patient no. 4 and 10) and one patient had a complete disruption of the brachial artery (case 1, patient no. 6; Fig. 1). Three patients had a distal radius fracture on the ipsilateral side.

**Table 1:** Demographics and clinical characteristics of patients with open elbow dislocations

Patient	Age (years)	Sex	Type of dislocation	Limb	Mechanism of injury	Type of fracture dislocation	Gustilo and Anderson type	Associated injury
1	39	M	Simple	L	Fall from height	NA	II	Ipsilateral distal radius fracture (23C3)
2	41	M	Simple	L	Fall from height	NA	II	NA
3	39	M	Simple	L	Fall from height	NA	IIIA	NA
4	45	F	Simple	L	Fall from height	NA	I	Radial nerve injury
5	53	M	Simple	R	Fall from height	NA	II	Ipsilateral distal radius fracture (23C3)
6	50	F	Simple	R	Fall from height	NA	IIIC	Brachial artery injury
7	55	M	Simple	R	Fall from height	NA	II	NA
8	53	M	Simple	L	Motor vehicle accident	NA	IIIA	NA
9	47	F	Complex	L	Motor vehicle accident	Distal humerus (13A3)/olecranon	IIIA	NA
10	24	M	Complex	R	Crush injury	Distal humerus (13C3)/Monteggia-like lesion	IIIC	Radial nerve injury
11	54	M	Complex	L	Motor vehicle accident	Distal humerus (13C3)/olecranon	IIIA	NA
12	60	M	Complex	L	Fall from height	Terrible triad injury	II	Ipsilateral distal radius fracture (23C1)
13	52	M	Complex	R	Fall from height	Terrible triad injury	II	NA
14	61	M	Complex	L	Fall from height	Distal humerus (13B3)	IIIA	NA
15	40	M	Complex	L	Motor vehicle accident	Monteggia-like lesion	II	NA
16	20	M	Complex	L	Motor vehicle accident	Monteggia-like lesion	I	NA
17	53	F	Complex	R	Fall from height	Monteggia like lesion	II	NA
18	56	M	Complex	L	Fall from height	Terrible triad injury	I	NA
19	83	F	Complex	L	Fall from height	Olecranon	IIIA	NA
20	73	F	Complex	R	Fall from height	Coronoid fracture type III	I	NA
21	38	M	Complex	L	Motor vehicle accident	Distal humerus (13C3)/olecranon	II	NA

*F* female, *M* male, *L* left, *R* right, *NA* not applicable

### Treatment protocol

Patients with simple elbow dislocations were treated with surgical debridement and open articular reduction. During primary surgery, patients with simple elbow dislocation were tested for varus and valgus instability with dynamic fluoroscopy. In seven out of eight patients with simple elbow dislocation, the collateral ligaments were reattached with anchors during the primary surgery according to the findings under fluoroscopic guidance (medial collateral ligament, *n* = 4; both collateral ligaments, *n* = 3). Two patients had additional temporary stabilization with an external fixator. One patient (case 1, patient no. 6) was treated with a temporary external fixator and the brachial artery was reconstructed using an autologous graft from the greater saphenous vein.

Complex elbow instabilities were treated according to the bone injury. Four out of 13 patients had primary stabilization with a temporal external fixator and underwent definite surgical treatment after 9–35 days (distal humerus/olecranon, *n* = 3; terrible triad, *n* = 1). Two out of 13 patients (both Monteggia-like lesion) underwent locked plating of the proximal ulna and osteosynthesis of the radial head with an additional temporary external fixator. The remaining seven patients underwent osteosynthesis during primary surgery (terrible triad, *n* = 2; distal humerus, *n* = 2; Monteggia-like lesion, *n* = 1; olecranon, *n* = 1; coronoid, *n* = 1) without additional external stabilization.

### Follow-up protocol

The functional outcome of the upper limb was determined using the Disability of Arm, Shoulder and Hand (DASH) outcome questionnaire. The range of motion (ROM) was measured in the elbow and forearm. Elbow function was evaluated using the Morrey Elbow Performance Score (MEPS) and wrist function was measured using the Mayo Wrist Score (MWS). The pain level was assessed using the Visual Analog Scale (0 = no pain, 10 = maximum pain) for both the elbow in rest (VAS_r_) and under pressure (VAS_p_). Complications and subsequent surgeries were also assessed.

### Statistical analysis

Mean and standard deviation (SD) were calculated for continuous variables. The Mann–Whitney *U* test was used in the analysis of different groups of patients. A two-tailed *p* value of <0.05 was considered to show a statistically significant difference. Fisher’s exact test was used in the analysis of contingency tables. Since the study was purely exploratory in design, and multiple tests without adjustment for multiplicity were performed, the reported *p *values can be interpreted only descriptively. SPSS (version 23.0; SPSS, Chicago, IL, USA) was used for the analysis.

## Results

**Fig. 2:**
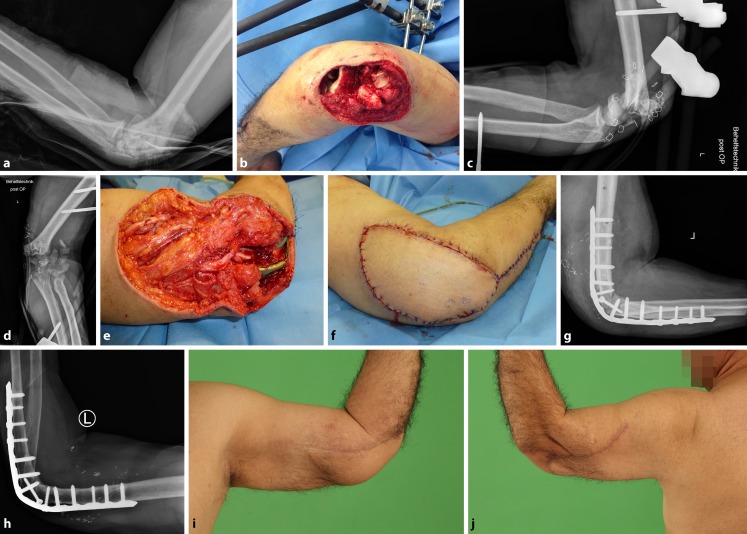
Case 2 (patient no. 11). **a** A 54-year-old patient had a traffic accident and sustained a complex open elbow dislocation with periarticular fracture on both sides of the elbow: distal humerus fracture (13C3/AO) and olecranon fracture. **b**–**d **Primary surgical treatment included wound debridement, open reduction, and temporary stabilization with an external fixator. **e**–**g **Before definitive surgical treatment, the patient had staged wound debridement and vacuum therapy 5, 10, and 15 days after primary surgery. After 19 days, the patient had definite surgical treatment with humero-ulnar arthrodesis at 90° of elbow flexion with a locking compression plate and soft tissue reconstruction with a free adipocutaneous perforator flap from the ipsilateral anterolateral thigh. **h**–**j **The ulnar nerve was revised 6 weeks later. At the 46-month follow-up, the patient complained of permanent ulnar nerve dysfunction. The patient achieved a favorable clinical outcome (DASH 15, MEPS 70, VAS_r_ 3) with a stable elbow

After a mean follow-up of 57 months (24–98 months), the mean DASH score was 20 ± 15, the MEPS averaged 82 ± 11 points, and the MWS was 74 ± 22 points. The mean VAS_r_ score was 1.3 ± 2.4 and the mean VAS_p_ score was 2.3 ± 1.4. The ulnohumeral arc of motion of the injured side was significantly decreased compared with the uninjured contralateral side (104°± 39 vs. 137°± 8; *p* = 0.001). Patients with I°/II° open elbow dislocations had a better clinical outcome according to the MEPS (86 ± 11 vs. 76 ± 9; *p* = 0.045) and a comparable outcome according to the DASH score (19 ± 18 vs. 21 ± 9; *p* = 0.238). There was no difference between simple and complex dislocation types in terms of the MEPS (86 ± 8 vs. 80 ± 13; *p* = 0.268) and the DASH score (16 ± 8 vs. 22 ± 18; *p* = 0.804). The ulnohumeral arc of motion in simple elbow dislocations was 117° ± 17. In the group of patients with complex elbow dislocations, two had surgical arthrodesis of the elbow. The other 11 patients achieved a comparable mean arc of ulnohumeral motion of 113° ± 20 (*p* = 0.840). Four out of 13 patients with complex elbow dislocations had periarticular fractures on both sides of the elbow, which was associated with a worse clinical outcome with a mean MEPS of 73 (range, 60–85); two of these patients had arthrodesis of the elbow (e. g., case 2; Fig. 2). Detailed results for each patient are shown in Table 2.

**Table 2:** Detailed results of patients with open elbow dislocations

Patient	Arc of ex/flex	Arc of pro/sup	MEPS	Complication	Revision (time from trauma to revision, days)
1	140	160	95	None	–
2	125	150	85	None	–
3	115	120	85	None	–
4	85	80	85	Postoperative radial nerve palsy	Neurolysis (5)
5	120	160	80	None	–
6	120	160	85	None	–
7	130	160	100	None	–
8	100	140	75	None	–
9	110	80	75	None	–
10	0	0	60	Pseudarthrosis after surgical arthrodesis	Re-osteosynthesis, bone graft (173)
11	0	80	70	Postoperative ulnar nerve entrapment	Neurolysis (54)
12	120	160	85	Postoperative ulnar nerve entrapment	Neurolysis (1740)
13	120	160	85	None	–
14	130	120	85	Postoperative median nerve palsy	Neurolysis (50)
15	70	80	60	Pseudarthrosis of the olecranon	Re-osteosynthesis, bone graft (178)
16	110	160	85	Pseudarthrosis of the olecranon	Re-osteosynthesis, bone graft (99)
17	130	100	75	Postoperative radial nerve palsy	Neurolysis (125)
18	130	100	100	Arthrofibrosis	Implant removal, joint release (177)
19	135	160	75	None	–
20	100	100	100	Postoperative ulnar nerve entrapment	Neurolysis (142)
21	90	140	85	Pseudarthrosis of the olecranon	Re-osteosynthesis, bone graft (377)

*MEPS *Mayo Elbow Performance Score, *ex* extension, *flex* flexion, *pro* pronation, *sup* supination

Overall, 11 patients (52%) had postoperative complications and 11 patients underwent at least one revision surgery. One patient with simple elbow dislocation had a postoperative palsy of the radial nerve that resolved after revision surgery with neurolysis of the radial nerve. After complex elbow dislocations, ten out of 13 patients (77%) had postoperative complications and underwent at least one revision surgery (simple vs. complex dislocation: 13% vs. 77%, *p* = 0.008). The most frequent complication in complex elbow dislocations was neurovascular problems (*n* = 5), for which revision surgery was performed. Three patients had implant-related postoperative ulnar nerve entrapment, which was revised via metal removal and neurolysis. One patient had postoperative palsy of the median nerve, and one patient suffered from transient palsy of the radial nerve after application of an external fixator. The palsy of the radial nerve resolved after revision of the distal humeral pin. Another four patients with complex dislocations had delayed fracture healing or pseudoarthrosis (olecranon, *n* = 3; arthrodesis, *n* = 1) and underwent re-osteosynthesis with autologous bone grafting. One patient underwent soft tissue reconstruction with a free adipocutaneous perforator flap from the ipsilateral anterolateral thigh. None of the patients in the current study suffered from a superficial or deep infection. Periarticular ossifications were observed in three of 21 patients (14%).

## Discussion

Open elbow dislocations represent a rare variant of elbow dislocations with little known about the treatment and the expected outcome in these patients. The most important finding of the current study is that a favorable outcome can be achieved after open elbow dislocations, with a mean MEPS of 82 ± 11 and a mean DASH score of 20 ± 15. The mean ulnohumeral arc of motion in simple elbow dislocations was 117° and in complex elbow dislocation 113° (excluding two patients with ulnohumeral arthrodesis). However, the surgeon should be aware of the high complication and revision rates after these types of injuries, especially in complex dislocations (ten out of 13 patients in this series). The most frequent complications in the current study were neurovascular problems (*n* = 6, 29%).

The findings of the current study are in agreement with the results of Boretto et al., who published a study with the largest case series (18 patients) of open elbow dislocations in 2014 [2]. Patients with simple and complex elbow dislocations had a mean arc of ulnohumeral motion of 117° and 110°, respectively. The mean Broberg and Morrey score was 90 points, and 12 out of 18 patients (67%) had complications. The most frequent complication was neurovascular problems (*n* = 9), which is also in agreement with the current findings. In 2009, Ayel et al. published a case series of nine patients with elbow dislocation and concomitant rupture of the brachial artery, of whom nine patients had an open elbow dislocation [1]. Revascularization was performed with a brachial–antebrachial shunt using a great saphenous graft or the ipsilateral basilic vein, and patients had a good clinical outcome with a mean MWS of 86 points. Other data in the literature on open elbow dislocations are rare and mostly related to case reports.

A large series of 136 combat-related open elbow fractures—a comparable type of injury—was published by Dickens et al. in 2015 [4]. The authors reported a mean MEPS of 68 (range, 30–100) with an average ulnohumeral arc of motion of 89°. A bipolar fracture pattern (fractures on both sides of the elbow) and more severe soft tissue injury (Gustilo and Anderson fracture type) were associated with decreased ROM and worse outcomes according to the MEPS, which is also in agreement with the findings of the current study.

Recently, our study group published the results of a large series of patients with closed elbow dislocations, both simple and complex, with a comparable follow-up period and rehabilitation protocol [10, 11]. mean MEPS of 94 ± 11 was achieved for simple elbow dislocations and the mean ulnohumeral arc of motion of the injured elbow was 135°. In all, 15 out of 118 patients (13%) had complications and nine patients (8%) underwent revision surgery. According to the results of the current study, patients with simple open elbow dislocations had a worse clinical outcome than did patients with closed simple elbow dislocations according to the MEPS (86 ± 8 vs. 94 ± 11) and the ROM (ulnohumeral arc of motion: 117° vs. 135°). Interestingly, in the current study only one out of eight patients (13%) with simple open elbow dislocations had a complication (radial nerve palsy) and underwent revision surgery with neurolysis, which is comparable to the results of simple closed elbow dislocations. Patients with closed complex elbow dislocations had a mean MEPS of 77 ± 17 and a mean ulnohumeral arc of motion of 114°, which is comparable to the results of the current study (MEPS: 80 ± 13; ulnohumeral arc of motion: 113°). However, patients with complex open dislocations had higher complications rates (77% vs. 42%) and underwent more revision surgeries (77% vs. 26%).

Summing up the results of the current study and the current knowledge from the literature, a satisfying clinical outcome can be achieved after both simple and complex open elbow dislocations. The treatment of open elbow dislocations is challenging and in some cases interdisciplinary management is necessary in the event of neurovascular damage or when soft tissue coverage is needed, such as in the two cases presented in the current study.

### Limitations

The present study is limited by its retrospective design and small sample size. There was no control group and a power analysis was not performed. Although all patients had open elbow dislocations, the complex dislocation cases in particular comprised heterogeneous injury types. However, it should be noted that the current study presents one of the largest series of patients with open elbow dislocation in the current literature.

## Practical conclusion

- A favorable clinical outcome can be achieved after treatment of open elbow dislocations.
- The surgeon should be aware that these injuries are prone to neurovascular damage.
- Complex dislocations are associated with high rates of complications and revision surgeries.
